# Enhanced Insecticidal Efficiency of Transgenic Bt Cotton Seed Following Application of Amino Acid Combinations

**DOI:** 10.3390/plants15091403

**Published:** 2026-05-04

**Authors:** Mingyu Ji, Eltayib. H. M. A. Abidallha, Xiang Zhang, Yuan Chen, Dehua Chen

**Affiliations:** 1 Jiangsu Key Laboratory of Crop Genetics and Physiology/Co-Innovation Center for Modern Production Technology of Grain Crops, Yangzhou University, Yangzhou 225009, Chinayzzhangxiang@163.com (X.Z.); cheny@yzu.edu.cn (Y.C.); 2 Department of Forest Management, Faculty of Forestry, University of Khartoum, Khartoum 13314, Sudan

**Keywords:** Bt cotton, amino acid treatment, cotton seed, Bt toxin, nitrogen metabolism

## Abstract

Low Bt toxin concentration in seeds results in low insecticidal efficacy in transgenic Bt cotton. In order to improve the insecticidal efficacy of seeds, two treatments with different amino acid combinations (5 amino acids comprising aspartic acid, glutamic acid, proline, methionine, and arginine; and 21 amino acids) were applied to two Bt cotton cultivars at peak boll stages in 2021 and 2022. The results showed that the amino acid treatments enhanced the seeds’ Bt toxin concentration by 13.5–34.2% compared with the untreated control in a two-year study. However, the difference for the Bt toxin was not significant between the two amino acid treatments. In the seeds, Bt toxin levels correlated positively with amino acid and soluble protein contents, as well as Glutamic-Pyruvic Transaminase (GPT) and Glutamate Oxaloacetate Transaminase (GOT) activities. Conversely, negative correlations were observed between the Bt toxin and the activities of protease and peptidase. Compared with the control, hazard boll rates were also reduced following application of the two amino acid combinations, while no difference was observed between the two amino acid treatments. Because the two treatments performed similarly, these results suggest that applying a simpler combination of five amino acids is an effective and efficient strategy for enhancing the insecticidal efficacy of cotton seeds.

## 1. Introduction

Bt transgenic cotton has been extensively cultivated because of its insecticidal properties [1,2]. The development of genetically modified cotton expressing Bt toxin offers many important economic and social benefits, including a reduced use of pesticides, decreased environmental pollution, savings in labor and cost, and an indirect increase in yield. Nevertheless, the insecticidal effect of Bt cotton varies widely across different stages of its growth, while also showing significant temporal and spatial characteristics [3,4]. The Bt toxin concentration also changes dynamically in the same organ throughout different developmental stages [5,6]. The Bt toxin content in cotton leaf is usually found to be higher than that in the boll, flower, and square [7,8,9]. The effectiveness of the Bt cotton in controlling insect pests is related to nitrogen metabolism, as Bt toxin is influenced by the processes of nitrogen metabolism [10,11]. Enhancement of the insecticidal efficacy in Bt cotton can be observed with the application of high levels of nitrogen fertilizer [12,13]. Previous research has shown that optimal nitrogen fertilization resulted in increased boll size and ultimately a greater cotton yield [14,15]. Recent studies have also reported that with elevated nitrogen input, most nitrogen was partitioned into cotton seeds rather than the boll shells of developing bolls, resulting in a decline in the Bt toxin content in the boll shell because of decreased protein synthesis and increased protein degradation [16]. A notable inverse relationship was observed between the concentration of Bt endotoxin in seeds and the content of Bt toxin in boll shells when inadequate nitrogen was available [17]. Hence, it is important to maintain a proper nitrogen balance between the seed and boll shell to ensure the insecticidal efficiency of Bt cotton [18]. Spraying exogenous amino acids is an effective way to regulate nitrogen metabolism in crops [19]. Recent findings [20] have shown that foliar application of exogenous amino acids onto soybean can directly activate and intervene in the initial steps of nitrogen assimilation in plants. This treatment not only promotes the nutrient absorption capacity of roots but also fundamentally improves the physiological, nutritional and metabolomic efficiency of nitrogen use and the final grain yield. Rossi et al. [21] found that amino acids play a dual role in gramineous field crops, serving both as carriers for nitrogen storage and as signaling molecules for stress defense. Spraying exogenous amino acids can prevent the physical and biochemical damage to the internal nitrogen metabolism system caused by adverse weather conditions, thereby ensuring the smooth transfer of photosynthetic energy to nitrogen assimilation processes [22]. Liu et al. [23] reported that if exogenous amino acids are available during the peak bloom stage, this could enhance the concentration of Bt toxin in cotton bolls and increase lint yield with the application of standard soil nitrogen fertilization; enhanced boll amino acid content was also detected at durations of 15 to 25 days after flowerings. These findings indicated that the use of nitrogen and amino acids could potentially impact the content of Bt toxin in Bt cotton. However, little is known about the influence of amino acid, especially for different amino acid combinations, on the Bt toxin content in cotton seeds. A previous study in 2017 investigated the leaf amino acid composition and contents under temperature stress at the square and boll stages, five amino acids, namely, aspartic acid, glutamic acid, proline, methionine, and arginine, were found to be the main components of Bt toxin according to this study [17]. Therefore, we hypothesized that external amino acid application, including the 5-amino acid and 21-amino acid combinations, would affect the Bt toxin concentration of the cotton seed; in addition, the associated mechanism of nitrogen metabolism was explored.

## 2. Results

### 2.1. Influence of Amino Acid Treatment on the Concentration of Bt Toxin of Cotton Seed

The Bt toxin content exhibited a temporal decline as the growth season progressed, with overall values at 15 DAF being significantly higher than those at 20 DAF and 25 DAF (Table 1). The application of amino acid combinations significantly altered the Bt toxin content in cotton cultivars Sikang1 (K1) and Sikang3 (K3) across various days after flowering (DAF). Analysis of variance revealed that the effect of treatment (T) was highly significant (*p* < 0.01) across all sampling days in both 2021 and 2022. The interaction between cultivar and treatment (K × T) was also significant (*p* < 0.05), whereas the cultivar main effect (K) was not significant.

The untreated control (CK) consistently maintained the lowest values throughout the sampling days. Exogenous amino acid application effectively elevated Bt toxin content. At 15 DAF in 2021, the K1T1 and K1T2 treatments recorded values of 315.2 and 312.1, representing increases of 10.6% and 9.5%, respectively, compared to K1CK. The Sikang3 cultivar exhibited a synchronized upward trend at this stage, with K3T1 and K3T2 showing increases of 10.5% and 12.3% relative to K3CK. This increase was also maintained in later developmental stages.

Multiple comparisons indicated that T1 (the 5-amino acid combination) and T2 (the 21-amino acid combination) showed no significant differences at the vast majority of time points. Thus, the application of the simpler 5-amino acid combination achieves an enhancement efficacy equivalent to that of the 21-amino acid combination.

### 2.2. Effects of Different Amino Acid Combinations on Nitrogen Metabolism in Cotton Seed

#### 2.2.1. Contents of Amino Acids and Soluble Proteins in Cotton Seed

**Figure 1:**
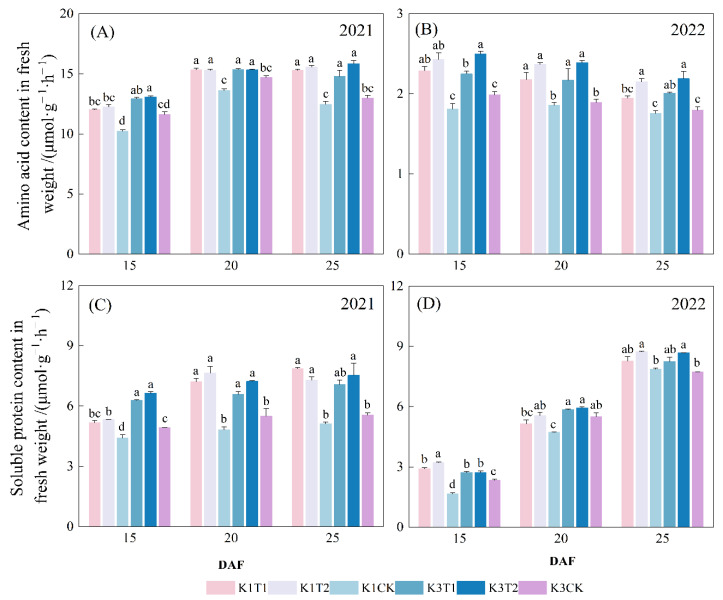
Influence of amino acid composition on: (**A**) amino acid content and (**C**) soluble protein content of the cotton seed in 2021. Influence of amino acid combination on: (**B**) amino acid content and (**D**) soluble protein content in cotton seed in 2022. Note: K1 and K3 represent cultivars Sikang1 and Sikang3; T1 and T2 represent treatments with combinations of 5 and 21 kinds of amino acid, respectively; CK, the control; DAF, days after flowering. Treatments labeled with the same letter are statistically not significantly different (least significant difference test at 0.05 level).

The amino acids and soluble protein content in the seed under two different amino acid combinations over the two-year study are shown in Figure 1. In 2021, the soluble protein content in cotton seed at 20 DAF increased under treatments T1 and T2 by 49.7% and 58.8% for K1, and by 19.8% and 31.5% for K3. In 2022, the soluble protein content of the cotton seed at 25 DAF increased under treatments T1 and T2 by 5.1% and 11.1% for K1, and by 6.9% and 12.4% for K3, respectively. However, no remarkable differences were observed for the soluble protein content of the seed between the two treatments with different amino acid combinations.

#### 2.2.2. GPT and GOT Activities in Cotton Seed

**Figure 2:**
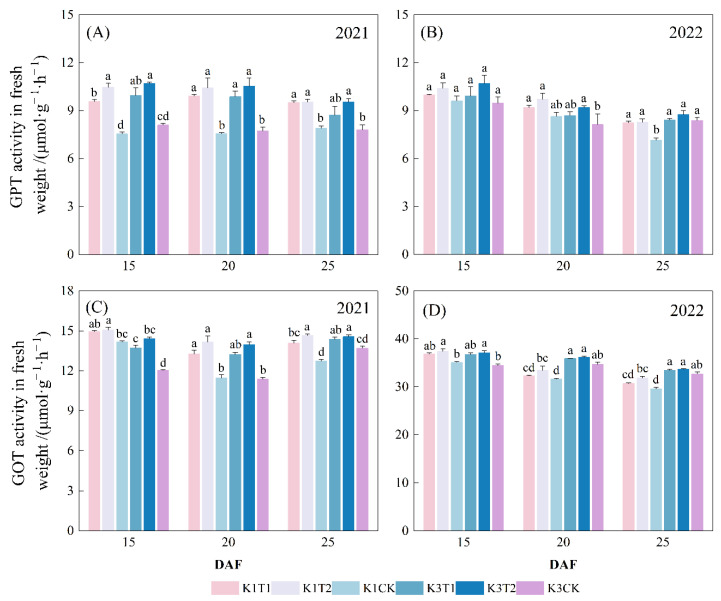
Effects of amino acid combination on: (**A**) GPT activity and (**C**) GOT activity in cotton seed in 2021. Influence of amino acid combination on: (**B**) GPT and (**D**) GOT activity in 2022. Note: K1 and K3 represent cultivar Sikang1 and Sikang3 respectively; T1 and T2 represent treatment with combinations of 5 and 21 kinds of amino acid, respectively; CK, the control; DAF, days after flowering. Treatments within the same year labeled with the same letter are statistically not significantly different (least significant difference test at 0.05 level).

The treatments with varied amino acid combinations led to a significant increase in GPT and GOT activities for both years (Figure 2). In 2021, GOT activity in cotton seed at 20 DAF increased for treatments T1 and T2 by 15.5% and 23.5% for K1, and by 16.2% and 22.7% for K3, respectively, compared with the control. In 2022, GOT activity in cotton seed for treatments T1 and T2 increased by 2.2% and 6.0% for K1, and by 3.5 and 4.3% for K3, respectively. Similar results were observed for the GPT activity of both studied cultivars in 2021 and 2022. However, no significant differences in GPT and GOT activities were detected between the two treatments with different amino acid combinations.

#### 2.2.3. Protease and Peptidase Activities in Cotton Seed

**Figure 3:**
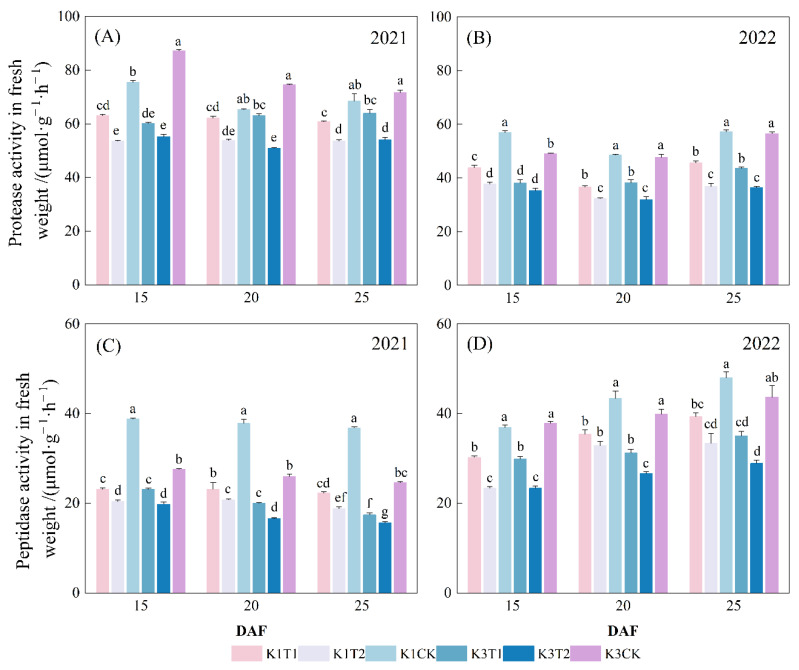
Influence of amino acid combination on: (**A**) protease and (**C**) peptidase activities of cotton seed in 2021. Influence of amino acid combination on: (**B**) protease and (**D**) peptidase activities of cotton seed in 2022. Note: K1 and K3 represent cultivars Sikang1 and Sikang3 respectively; T1 and T2 represent treatments with combinations of 5 and 21 kinds of amino acid, respectively; CK, the control; DAF, days after flowering. Treatments within the same year labeled with the same letter are statistically not significantly different (least significant difference test at 0.05 level).

Compared with the control, the two treatments with varied amino acid combinations resulted in significantly reduced activities of protease and peptidase in the cotton seed in both years (Figure 3). Specifically, in 2021, protease activity in cotton seed at 20 DAF declined for treatments T1 and T2 by 5.0% and 17.9% for K1, and by 15.7% and 32.0% for K3, respectively. In 2022, protease activity in the cotton seed at 20 DAF declined with treatments T1 and T2 by 24.5% and 33.5% for K1, and by 19.7% and 33.0% for K3, respectively. The peptidase activities of the seed showed similar results with the application of two different amino acid combinations. However, protease and peptidase activities did not differ significantly between the two treatments with different amino acid combinations.

### 2.3. Relationship Between Bt Toxin Concentration and Parameters Related to Nitrogen Metabolism in Cotton Seed

**Figure 4:**
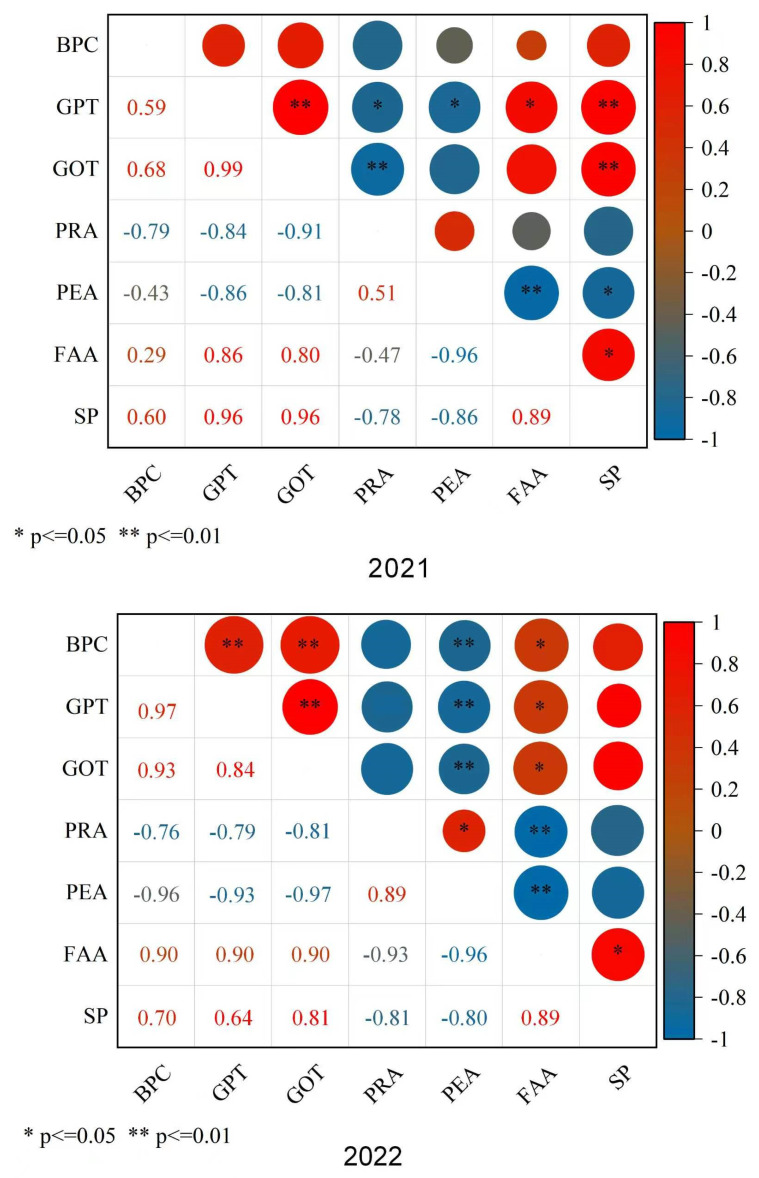
Correlation analysis between the Bt toxin level and parameters related to nitrogen metabolism in cotton seeds. BPC, Bt protein concentration; GPT, GPT activity; GOT, GOT activity; PRA, protease activity; PEA, peptidase activity; FAA, amino acid content; SP, soluble protein content. * and ** indicate that the correlation coefficient reached a significant level at α = 0.05 and α = 0.01, respectively.

A strong relationship was observed between the concentration of Bt insecticidal toxin and the activity of enzymes in the cotton seed (Figure 4). In both 2021 and 2022, significant positive correlations were observed between Bt toxin content and the activities of GOT and GPT in cotton seed. In contrast, both protease and peptidase activities were negatively correlated with the Bt toxin content of cotton seed. Furthermore, a notable positive relationship was observed between the Bt toxin content and the amino acids and soluble protein content in both years. The results showed that enhanced protein synthesis and declined protein decomposition were related to enhanced Bt toxin content in cotton seeds.

### 2.4. Effects of Five and Twenty-One Kinds of Amino Acid Combination on the Insecticidal Effect of Bt Cotton Seeds

The number of bollworms and the hazard boll rate were significantly reduced under the treatments with different amino acid combinations, while boll Bt toxin levels were enhanced in both the 2021 and 2022 field studies (Table 2). In 2021, the number of bollworms were 0.9and 1.1, 0.8 and 0.7 per 10 plants under the two treatments with different amino acid combinations for cultivars Sikang1 and Sikang3, respectively. Conversely, the bollworms were 2.7 and 3.2 per 10 plants under the control treatments for cultivars K1 and K3, respectively. However, no notable difference was found in the bollworms per 10 plants. Similar results were observed in 2022. Compared with the control, hazard boll rates were also reduced following application of the two amino acid combinations.

## 3. Discussion

### 3.1. The Primary Amino Acid Combination of the Bt Toxin Can Significantly Enhance Bt Insecticidal Efficacy

Recently, studies have observed that spraying 10 µM L^−1^ glutamic acid on rice markedly alleviated oxidative stress under adverse conditions [24]. Glutamic acid directly upregulated the activities of NR, NiR, GS, and GOGAT enzymes in the nitrogen assimilation pathway and promoted endogenous proline metabolism. This indicates that exogenous glutamic acid serves not only as a basic substrate for nitrogen metabolism but also as a signaling molecule that accelerates nitrogen assimilation and stress resistance responses in crops [25,26]. Moreover, aspartic acid and glutamic acid exhibit a pronounced synergistic effect in promoting nitrogen assimilation and carbon–nitrogen balance, making them excellent composite biostimulants [27]. Exogenous amino acid supplementation provides plants with a “shortcut” for synthesizing organic nitrogen compounds, which can not only fine-tune nitrogen metabolism but also prevent plants from consuming excessive energy through nitrate reduction under stress [28,29]. In cotton, it has also been demonstrated that amino acid supplementation can enhance leaf Bt toxin levels in cotton [17,30]. Nitrogen can influence the Bt toxin content of leaf and reproductive organs, and can increase leaf insecticidal efficacy [13,31]. In the present study, the 5 and 21 amino acid combinations significantly increased the Bt toxin concentration in cotton seed; however, the two amino acid treatments did not differ significantly in their effect on Bt toxin levels of the cotton seed. The results were proven further by the changes in the number of bollworms and the hazard boll rate with the application of the two amino acid combinations. These findings indicated that application of the 5-amino acid combination at the peak of the flowering period was of more practical value because of the increased cost of the application of the 21-amino acid combination. The significant cultivar*amino acid treatment interaction (*p* < 0.05) indicates that the response of different cotton cultivars to exogenous amino acid applications is not uniform. The magnitude of the physiological enhancement depends heavily on the genetic background of the specific cultivar. While both Sikang1 and Sikang3 benefited from the treatments, their distinct biological traits led to differential response patterns. Therefore, it was suggested that the application of the main five-amino acid combination constituting CryIAc protein was more practical for increasing the Bt insecticidal efficacy.

### 3.2. Increased Protein Compounding and Decreased Protein Decomposition Resulted in Increments of Bt Toxin Concentration

Maintaining the insecticidal efficacy of transgenic Bt cotton throughout its growth cycle remains a critical challenge, as environmental stresses and natural aging often lead to a spatiotemporal decline in Bt toxin expression [32,33]. Previous studies have shown that protein synthesis and decomposition affected the Bt toxin content of cotton seed [17,30,34]. It was found that an increase in the leaf protease and peptidase activities resulted in a marked reduction in Bt insecticidal efficacy with the application of ETDA [35,36]. It has also been reported that an increase in aspartic acid, glutamic acid, pyruvate, and arginine enhanced the Bt toxin content with mepiquat chloride application [23,37]. In our present study, GPT and GOT activities, soluble protein, and amino acid content were enhanced, while protease and peptidase activities declined in cotton seed following application of the two kinds of amino acid combinations. The findings indicated that reduced protein decomposition and bolstered protein compounding in cotton seed resulted in an enhanced Bt toxin concentration. These findings were further proven by the significant positive correlations observed between GOT and GPT activities with the Bt toxin level, along with a markedly negative correlation between protease and peptidase activities with the Bt toxin concentration, a notable positive relationship between the amino acids and soluble protein content with the Bt toxin content. These findings were also consistent with earlier studies [9,16,18]. Therefore, enhanced protein combination and reduced protein decomposition were the primary drivers of the increase in the Bt toxin level with application of the two amino acid combinations.

## 4. Materials and Methods

### 4.1. Materials and Field Experimental Arrangement

The studies were carried out during the 2021–2022 cotton growing seasons, Yangzhou University Farm, Jiangsu Province, China. A split-plot arrangement was used with three replications. The conventional Sikang1 (K1) and the hybrid Sikang3 (K3) cultivars, which are widely grown in China, served as the main plot treatments. In this study, planting densities of 37,500 and 27,000 plants per hectare were imposed for K1 and K3, respectively. The subplots were treated with three distinct treatments with different combinations of amino acids, including the untreated control (CK), a 5-amino acid combination constituting the main component of Bt toxin (aspartic acid, glutamic acid, proline, methionine, arginine) (T1), and a 21-amino acid combination (T2). The concentration of applied amino acids was 20 mg kg^−1^. Water spraying was used as the untreated control treatment (CK). The treatments were implemented at the peak of the blooming period.

Seedling-raising occurred on 3 April 2021 and again on 6 April 2022 in a greenhouse; plants were transplanted to the field on 15 May 2021 and 17 May 2022. The soil contained 20.5 and 20.1 g·kg^−1^ of organic matter, and the available N, P, and K in 2021 and 2022 were 109.5 and 111.1, 20.6 and 19.5, and 84.6 and 84.9 mg·kg^−1^, respectively. Fertilizers, irrigation, mepiquat chloride and insecticides, were applied according to local culture practices. Each experimental plot was 16 m in length, 8.0 m in width, and 0.83 m in row spacing.

### 4.2. Preparation of Plant Material

#### 4.2.1. Sampling

The samples were harvested at 15, 20 and 25 days after anthesis; five bolls were harvested from the first position of the eleventh to thirteenth fruiting branches. The cotton seed was separated from each boll and mixed evenly for five bolls. Three separate subsamples from each plot, weighing 0.3 g fresh weight for each sample, were used for the measurement of Bt toxin content and related compounds and enzyme activity.

#### 4.2.2. Bt Toxin Content

Enzyme-linked immunosorbent assay (ELISA) was used to determine the Bt toxin content of the cotton seed. Details of the method are described by Liu et al. [23].

#### 4.2.3. Free Amino Acid and Soluble Protein Content

Following the method of Wu et al. [38], near-infrared reflectance spectroscopy was used to quantify the overall content of free amino acids. Total soluble protein content was determined using the Coomassie Blue dye-binding assay described by Bradford [39].

#### 4.2.4. Glutamic-Pyruvic Transaminase (GPT) and Glutamate Oxaloacetate Transaminase (GOT)

The activities of GOT and GPT were measured according to the Reitman–Frankel colorimetric method [40]. Seed samples (0.2 g FW) were homogenized in a pH 7.2 Tris-HCl buffer, followed by centrifugation to collect the supernatant for enzyme assays. For GOT assay, the supernatant was reacted with alanine, pyridoxal phosphate, and 2-oxoglutarate at 37 °C, then the reaction was stopped with trichloroacetic acid and absorbance measured at 520 nm. The GPT assay used an identical procedure except that aspartate buffer replaced alanine in the reaction mixture.

#### 4.2.5. Protease and Peptidase Activities

Samples of the seeds (0.8 g) were homogenized at 4 °C in 1 mL of β-mercaptoethanol extraction buffer (a mixture of ethylene glycol, sucrose, and phenyl methyl sulfonyl fluoride, pH 6.8). This liquid was gathered for the purpose of assessing the protease activity of the samples. An assay of protease and peptidase activity was conducted according to the method described by Liu et al. [23].

#### 4.2.6. Bollworm and Damage Survey

In each plot, 10 consecutive cotton plants were selected from the middle two rows. Each plant was examined for all leaves, buds, flowers, and bolls; the number of larvae at different instars was recorded, and the hazard boll rate for each treatment was recorded and calculated. Hazard boll rate (%) = (Number of infested plants/Total number of plants surveyed) × 100%. During the survey, a plant is recorded as infested if any bollworm larvae, boreholes, or fresh frass are found on it.

### 4.3. Statistical Analysis

Prior to statistical testing, all data were subjected to the Shapiro–Wilk test to verify the normality of distribution and Levene’s test to confirm the homogeneity of variance. Proc ANOVA in SPSS30 was used to analyze the experimental data. The least significant difference test was used to evaluate the statistical significance among treatments. The Pearson correlation was used to calculate the degree of correlation.

## 5. Conclusions

The present study shows that the external use of different combinations of amino acids could enhance the insecticidal protein content (13.5–34.2%) in cotton seed. The application of the total amino acid combination may increase Bt toxin content slightly, but no notable distinctions were observed with the application of the combination of five amino acids constituting the Bt toxin in cotton seed. Enhanced protein synthesis and suppressed protein degradation were the main causes of the changes in the Bt toxin concentration in cotton seed.

## Figures and Tables

**Table 1:** Influence of the amino acid combinations on the Bt toxin content of cotton seed (ng·g^−1^ FW).

Treatment	2021			2022		
	DAF					
	15	20	25	15	20	25
K1CK	284.9 ± 9.12 d	179.1 ± 5.73 c	194.9 ± 8.77 d	129.0 ± 2.97 bcd	119.3 ± 3.82 de	114.1 ± 4.22 d
K1T1	315.2 ± 14.81 c	178.2 ± 5.35 ab	221.2 ± 6.86 b	139.8 ± 3.50 ab	139.8 ± 3.63 abc	134.8 ± 6.07 ab
K1T2	312.1 ± 11.55 c	193.5 ± 8.71 a	230.8 ± 7.39 b	149.1 ± 7.75 a	146.6 ± 6.74 a	141.4 ± 5.94 a
3CK	413.1 ± 10.74 b	176.4 ± 4.41 c	230.1 ± 10.81 c	121.9 ± 2.68 d	114.6 ± 2.87 e	110.0 ± 3.85 d
3T1	456.5 ± 18.26 a	174.3 ± 4.71 ab	258.7 ± 9.05 a	136.3 ± 5.04 abc	136.3 ± 6.13 bc	127.3 ± 5.09 bc
K3T2	463.9 ± 24.12 a	192.9 ± 6.94 a	261.6 ± 12.82 a	150.9 ± 7.09 a	145.5 ± 7.71 ab	136.4 ± 6.41 ab
ANOVA						
K	ns	ns	ns	ns	ns	ns
T	**	**	**	**	**	**
K × T	*	*	*	*	*	*

Note: K1 and K3 represent cultivars Sikang1 and Sikang3, respectively; T1 and T2 represent treatments with combinations of 5 and 21 amino acids, respectively; CK is the untreated control. Note: DAF, days after flowering; ANOVA, analysis of variance; ns, not significant. “*” and “**” are least significant difference tests at 0.05 and 0.01 levels respectively.

**Table 2:** Effects of the application of 5 and 21 kinds of amino acid combinations on insecticidal efficacy of Bt cotton seed.

	2021		2022	
Treatment	Bollworms/10 Plants	Hazard Boll Rate (%)	Bollworms/10 Plants	Hazard Boll Rate (%)
K1CK	3.2 ± 0.07 a	25.6 ± 0.67 a	3.5 ± 0.07 a	27.3 ± 0.93 a
K1T1	0.9 ± 0.01 bc	10.7 ± 0.28 bc	1.4 ± 0.04 bc	13.5 ± 0.41 bc
K1T2	1.1 ± 0.03 bc	10.3 ± 0.31 bc	1.2 ± 0.02 bc	12.8 ± 0.31 bc
K3CK	2.7 ± 0.05 a	22.4 ± 0.65 a	2.9 ± 0.07 a	26.1 ± 0.94 a
K3T1	0.8 ± 0.03 c	8.7 ± 0.23 c	1.1 ± 0.02 c	11.7 ± 0.30 c
K3T2	0.7 ± 0.02 c	8.2 ± 0.30 c	0.9 ± 0.02 c	10.8 ± 0.29 c

Note: K1 and K3 represent cultivars Sikang1 and Sikang3 respectively; T1 and T2 represent treatments with a combination of 5 and 21 kinds of amino acid, respectively; CK, the control. Differences between treatments within the same column with the same letter are statistically not significant (least significant difference test at 0.05 level).

## Data Availability

The original contributions presented in this study are included in the article. Further inquiries can be directed to the corresponding author.
